# Artificial urinary sphincter implantation: an important component of complex surgery for urinary tract reconstruction in patients with refractory urinary incontinence

**DOI:** 10.1186/s12894-018-0314-y

**Published:** 2018-01-08

**Authors:** Fan Zhang, Limin Liao

**Affiliations:** 1 Department of Urology, China Rehabilitation Research Center, Beijing, 100068 China; 2 Department of Urology, Capital Medical University, Beijing, China

**Keywords:** Artificial urinary sphincter, Urinary tract reconstruction, Refractory urinary incontinence, Outcomes

## Abstract

**Background:**

We review our outcomes and experience of artificial urinary sphincter implantation for patients with refractory urinary incontinence from different causes.

**Methods:**

Between April 2002 and May 2017, a total of 32 patients (median age, 40.8 years) with urinary incontinence had undergone artificial urinary sphincter placement during urinary tract reconstruction. Eighteen patients (56.3%) were urethral injuries associated urinary incontinence, 9 (28.1%) had neurogenic urinary incontinence and 5 (15.6%) were post-prostatectomy incontinence. Necessary surgeries were conducted before artificial urinary sphincter placement as staged procedures, including urethral strictures incision, sphincterotomy, and augmentation cystoplasty.

**Results:**

The mean follow-up time was 39 months. At the latest visit, 25 patients (78.1%) maintained the original artificial urinary sphincter. Four patients (12.5%) had artificial urinary sphincter revisions. Explantations were performed in three patients. Twenty-four patients were socially continent, leading to the overall success rate as 75%. The complication rate was 28.1%; including infections (*n* = 4), erosions (*n* = 4), and mechanical failure (*n* = 1). The impact of urinary incontinence on the quality of life measured by the visual analogue scale dropped from 7.0 ± 1.2 to 2.2 ± 1.5 (*P* <0.001).

**Conclusions:**

The primary sources for artificial urinary sphincter implantation in our center are unique, and the procedure is an effective treatment as a part of urinary tract reconstruction in complicated urinary incontinence cases with complex etiology.

## Background

Artificial urinary sphincter (AUS) implantation is a well-established treatment for refractory stress urinary incontinence (SUI) resulting from intrinsic sphincter deficiency (ISD) [1]. It is versatile and effective in a wide range of situations, including post-prostatectomy incontinence (PPI) or other urethral surgery-related incontinence, traumatic urethral disruption as a result of a prior pelvic fracture, radical pelvic surgery, neurogenic causes, and as a salvage procedure after other treatments have failed [2, 3].

AUS placement is primarily performed in men with post-radical prostatectomy (RP) incontinence [4]; there is scant published data for other etiologies [1–3, 5]. Interestingly, the majority priority cases for AUS implantation in our institute have been incontinence secondary to urethral injuries and neurogenic cases. A series of urinary tract reconstruction (UTR) might be needed combining AUS placement and other additional procedures in complex cases [3]. The type of injury, possibility of a previous failed repair, relatively restricted surgical access, or urethral stricture, together with inherent detrusor-sphincter dysfunction, make UTR more complicated [6]. Thus, the quality and choice of management modalities should be tailored to the unique needs of each individual. The purpose of this study was to present our experience of AUS implantation as a part of UTR for patients with complex and refractory urinary incontinence (UI).

## Methods

### Subjects

With the approval of the Ethics Committee of the China Rehabilitation Research Center, we reviewed 32 patients (31 males and 1 female) who underwent AUS placement (AMS 800; American Medical Systems, Minnetonka, MN, USA) for the treatment of UI secondary to different causes (April 2002 to May 2017). Written informed consent for participation was obtained from all participants in the study. All participants were adults and the mean age was 40.78 ± 16.58 years. Eighteen patients (56.3%) had pelvic fracture-associated urethral injuries (PFUI) (car accident or fall down) that were initially treated elsewhere and presented to us 8 months to 30 years after the injury with intractable incontinence, nine of whom developed urethral stricture after the Bank’s method and had recurrent contractures after initial management; two underwent male slings surgeries but had recurrent incontinence. Nine patients (28.1%) had neurogenic bladder (NB) dysfunction (meningomyelocele, 5; spinal cord injury, 4) and 5 (15.6%) underwent prostatectomies (Table 1). The consent to publish these information (age and detailed medical history) was obtained from all participants.

**Table 1:** Clinic characteristics of the patients treated with an artificial urinary sphincter (*n* = 32)

Characteristics	Value
Number of patients Age (yrs) Mean follow-up time (yrs)	32 40.78 ± 16. 58 3.3 ± 0.7
Type UI	
PFUI NB TURP RP	18 (56.3%) 9 (28.1%) 3 (9.4%) 2 (6.2%)
Number of previous surgeries for UI	
0–1 2 3 ≥ 4	10 (31.3%) 5 (15.6%) 5 (15.6%) 12 (37.5%)
Cuff size	
4 cm 4.5 cm 8 cm	21 (65.6%) 10 (31.3%) 1 (3.1%)*
Operative approach	
transperineal trans-scrotal trans-retropubic transcorporal (intracavernous)	16 (50%) 15 (46.9%) 1 (3.1%) 1*

Values are presented as the mean (±standard deviation) or number (%)

*PFUI* pelvic fracture-associated urethral injuries, *NB* neurogenic bladder, *TURP* transurethral resection of the prostate, *RP* radical prostatectomy. *8 cm cuff was implanted on the female case. *One transperineal case had a transcorporal approach implantation when revised

The pre-implantation evaluation comprised a clinical interview, surgical history, analysis of voiding diaries (time and voided volumes, pad changes, and UI episodes), physical examination, urinalysis, and urine culture. More invasive testing included urethrography and video-urodynamic assessment. Cystourethroscopy was required to verify urethral integrity, bladder neck patency, and vesicourethral anastomotic strictures. Ultrasound was routinely used to assess the upper urinary tract (UUT) and post-void residual urine. The UUT was also evaluated by magnetic resonance urography (MRU).

### Surgical technique

According to patients’ individual condition, the following operations were conducted before AUS placement, including two strictures incision, three sphincterotomies, and five urethral dilations in urethral injuries associated UI patients due to the refractory urethral stricture post Bank’s method; four patients with neurogenic UI underwent sphincterotomies or urethral dilations out of detrusor sphincter dyssynergia (DSD), 3 subsequently had augmentation cystoplasties. A urethral stricture was incised in one PPI patient. These surgeries were performed 3–6 months prior to AUS implantation as staged procedures in the series of UTR (Table 2).

**Table 2:** Patient operative history and treatment measures before AUS implantation

Number of patients (*N* = 32)		Etiology	Previous procedures (Times)	Least treatment to AUS interval (Yrs)	Our treatment before AUS	Complications	Management
1		PFUI	Upl,Spl,USD,USI	21	USD	none	none
2		PFUI	Upl,RV	0.5	cystoscopy*	none	none
3		NB	spondylolysis	none	cystoscopy	dysuria	revision
4		NB	Cty	3	Sty + AC	infection, erosion	explantation
5	1st time	NB	Cty	1	Sty	RI	revision explantation
	2nd time		AUS(RI)	0.5	cuff removal	infection	
6		PFUI	Upl,Spl,USD,USI	2	Sty	RI, erosion	explantation
7	1st time	PFUI	Upl,USI(2),MS	10	USD	erosion, infection	revision
	2nd time		AUS (erosion)	3	Cuff removal	Transcorporal implantations	none
8		PFUI	Upl,USD,USI	0.7	USD	none	none
9		NB	spondylolysis	13	USD,AC	none	none
10		PPI	TURP,USD	2	cystoscopy	none	none
11		NB	Spondylolysis	20	cystoscopy	none	none
12		PFUI	Upl,Spl,USI(2)	20	USD	none	none
13		PFUI	Upl,Spl,USD	1	USI	none	none
14		PPI	TURP,Upl,USI(2)	20	USI	none	none
15		PFUI	Upl,Spl,USI	3	Sty	none	none
16		PFUI	Upl,USI(2),US,MS	14	USI	none	none
17		PFUI	Upl,USI(2),Cty	1	USD	none	none
18		NB	Upl,Spl,USD	9	USD + AC	none	none
19	1st time	PFUI	Upl,USI	10	cystoscopy	fluid leakage	revision
	2nd time		AUS(mechanical failure)	one month	device removal	none	none
20		PFUI	Upl,Spl,USD,USI(2)	30	Sty	none	none
21		PFUI	Upl,USD,USI	18	cystoscopy	none	none
22		PPI	RP	3	cystoscopy	none	none
23		PPI	TURP,USD	3	cystoscopy	none	none
24 25 26 27 28 29 30 31 32		PFUI NB PFUI NB PFUI PFUI NB PFUI PPI	USD,USI Upl,USI(2),US,USD Upl USD Upl,USD(2),USI,Spl Upl,Spl,USI(2) USD Upl RP	11 36 2 1 10 2 2 1 1	cystoscopy cystoscopy cystoscopy cystoscopy cystoscopy cystoscopy Sty cystoscopy cystoscopy	none none none none none none none none none	none none none none none none none none none

Cystoscopy was routinely performed to identify the severity of urethra strictures. If the stricture was asymptomatic or not progressive within at least 12 months, and if the post-void residual volume (< 50 ml) and Qmax were acceptable, then the AUS device was implanted with the maintenance of the current stricture. *PFUI* pelvic fracture-associated urethral injuries, *NB* neurogenic bladder, *PPI* post-prostatectomy incontinence, *Upl* urethroplasty, *Sty* sphincterotomy, *Spl* sphincteroplasty, *Cty* cystostomy, *RV* reconstruction of the vagina, *RI* recurrent incontinence, *USD* urethral stricture dilation, *USI* urethral stricture incision, *AC* augmentation cystoplasty, *MS* male sling, *TURP* transurethral resection of the prostate, *RP* radical prostatectomy, *US* urethral stent

AUS implantation was performed as the standard procedure [2. 3]. Sixteen patients (50%) had the transperineal approach (two incisions) and 15 (46.9%) had the advanced trans-scrotal approach (one incision); based on individual local skin conditions. The female case had a trans-retropubic approach. Intravenous antibiotics (ceftriaxone and vancomycin) were given within 12 h prior to AUS surgery. Under general or spinal anesthesia, all patients were placed in the lithotomy position and the perineum was generously prepped with an alcohol and Betadine solution. Then, a Foley catheter with 8F–16F was inserted into the bladder. The AUS cuffs were placed at the bulbar urethra for the male cases and the bladder neck for the female case. The pump and balloon were implanted in the scrotum or labium majus and abdomen, respectively. The balloon pressure was 61–70 cm H_2_O in all cases. The indwelling catheter remained in situ for the first 24 h, then switched to an external urine collection device before system activation (4–6 weeks post-operatively). Antibiotic prophylaxis was maintained 3 days post-operatively, followed by 2 weeks of oral ciprofloxacin. The length of hospital stay ranged from 5 to 7 weeks, including 1 week of pre-operative evaluation and 4–6 weeks post-operative in-patient time for patients from remote area. The local patients usually discharge from hospital 1 week after operation and visit out-patient clinic for system activation.

### Assessment

All patients were assessed after AUS activation, followed by visits at 6 and 12 months, and by telephone interviews annually, or more frequently if needed.

The continence outcomes were assessed according to daily pad use. The impact of the UI in the patient’s quality of life (QoL) was evaluated with a visual analogue scale (VAS). Specifically, the patients indicated on a numeric scale the impact of UI on QoL. Numbers 1–10 represented mild-to-severe impact on QoL. Efficacy and safety results were conducted on the following end points: infection/erosion rates; explantation rate (defined as complete removal of the whole device); and dry rate (defined as the proportion of patients wearing no pads). Patients were considered to have surgical success if they were socially continent (one pad per day or less). Failure was defined as that caused by any reason, such as mechanical failure, surgical revision, or removal.

### Statistical analysis

Differences in durability according to different criteria were analyzed using the Student t-test. *P*-values <0.05 were considered significant. SPSS (version 17; SPSS, Inc., Chicago, IL, USA) was used for all analyses.

## Results

All AUS procedures were performed successfully. The devices were activated in 31 cases at 1-month after surgery; patient 4 had device explantation because of an immediate infection due to scrotal skin erosion. At the time of adjustment, patient 3 had acute urinary retention and patient 5 had refractory incontinence (RI), both underwent cuff revision. The other early post-operative complications included two cases of transient perineal pain and one case of scrotal hematoma, all of which were managed conservatively. For initial evaluation, the success rate was 90.6% (29/32), 93.5% (29/31) patients had social continence including 80.6% (25/31) completely dry.

At 6-month follow-up, patient 5 had device explantation due to urethral atrophy induced RI and chronic infection; patient 19 had device revision due to balloon perforation. The success rate with original device was 90.3% (28/31); the success rate with revision was 96.8% (30/31). At 12-month follow-up, the success rate was maintained.

At 24-month follow-up, patient 6 had device explantation due to urethral erosion and chronic infection, leading to success rate 96.7% (29/30).

At 36-month follow-up, patient 7 had transcorporal single device (cuff) revision due to urethral erosion. Three cases had favorable outcomes in the first 2 years and then experienced descending efficacy due to recurrent incontinence (RI) (patient 2. 7 and 20), leading to success rate 89.7% (26/29).

**Fig. 1:**
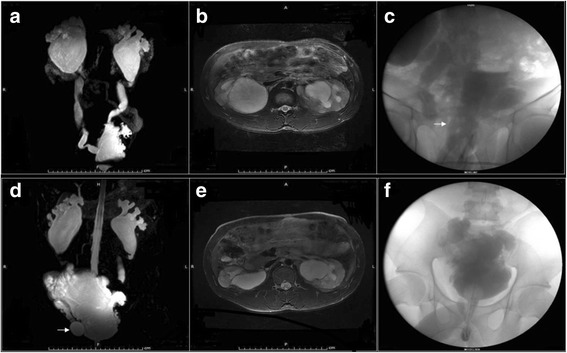
Magnetic resonance urography and cystogram assessment before and after AUS implantation. Hydronephrosis (**a,b**) and vesicoureteral reflux (**c** represented by the white arrows) were presented before operation. Upper urinary dilation was ameliorated after augmentation cystoplasty and AUS surgery (**d** the white arrows represent the pressure regulating balloons, **e**) and reflux was cured (**f**) in 6-month follow up

The mean follow-up time was 39 months. At the latest follow-up, 25 patients maintained the original AUS. Four patients had AUS revisions. Explantations were performed in three patients. Twenty-four patients were socially continent, leading to the overall success rate as 75% (24/32), and 15 out of the 24 patients were completely dry 46.9% (15/32). The success rates were 77.8% (14/18), 66.7% (6/9) and 80% (4/5) in PFUI cases, NB cases and PPI cases, respectively. The daily pad count dropped from 3.6 ± 1.5 to 1.2 ± 0.2 pads per day (P <0.001; Table 3). The impact of UI on the QoL measured by the VAS dropped from 7.0 ± 1.2 to 2.2 ± 1.5 (P <0.001). Patients had preserved UUT function in the series of UTR; a typical case is shown in Fig. 1.

**Table 3:** Functional outcomes of pad use in patients with AUS device during post-operative period

Interval (months)	pad use (*n* = 29)^a^			
	none	1 pad	2–3 pads	> 4 pads
< 24	8	6	1	0
24–48	4	2	2	0
>48	3	1	1	1

At the end of the follow-up period, 24 patients were shown to be socially continent and 15 patients were completely dry. The daily pad count dropped from 3.6 ± 1.5 to 1.2 ± 0.2 pads per day (P <0.001). ^a^Explantations were performed in three patients

## Discussion

AUS implantation has been the standard treatment for refractory SUI in males caused by sphincter deficiency. The quantity and level of evidence is 2b, as per the European Association of Urology guidelines [7]. Most information is gained from older case series after RP [8]. In the present study, the primary patients’ source is urethral injuries associated UI with complex treatment experiences, which was quite different from previous literature [1–9]. Our success rate (75%) was comparable to the recent critical systematic review (61% and 100%) [9]. Despite known complications, the patient satisfaction rates remained favorable. There were several notable findings from the current study that merit further discussion.

The mechanism of urethral injuries associated UI may be related to the original trauma or as a complication of transpubic surgery and damage to the associated nerves or sphincter. A previous study indicated that the development of incontinence could be related to the trauma itself, rather than the method of initial management [10]. In our series, the majority of patients had undergone at least two urethral surgeries with recurrent incontinence. In our opinion, the subsequent urethral repair procedures, especially improper incision of urethral stricture, may contribute to the development of UI.

The use of AUS for urethral injuries associated UI has been limited because of functional urethral length, surrounding fibrosis, urethral strictures, and distorted anatomy of the pelvis. Scarring caused by previous urethral surgical procedures may lead to more difficult dissection. A decrease in functional urethral length may potentially reduce the efficacy of AUS surgery. Therefore, it is essential to confirm the status of the urethra and identify concomitant anastomotic strictures before AUS placement. If an anastomotic stricture is refractory or progressive, it is necessary to treat the stricture first. We prefer excising strictures narrower than 20-Fr, despite transient worsening of incontinence, and ensure an adequate recurrence-free period. The length of the period depends on individual conditions. If the stricture is asymptomatic or not progressive for at least 12 months and patients have acceptable post-void residual volumes (<50 ml), we suggest implanting the AUS device with maintenance of the current stricture, since aggressive excision may worsen a stable urethra.

NB dysfunction with UI secondary to ISD is also an indication for AUS implantation [3]. NB patients may suffer from low bladder outlet resistance, and the AUS can offer such patients the possibility of spontaneous voiding. In the present study, two NB patients had cuffs easily and effectively placed. Four patients with detrusor overactivity and DSD had sphincterotomy or urethral stricture dilations, three of whom subsequently had augmentation cystoplasty before AUS implantation. Only one patient with pre-existing renal insufficiency and urethral stricture had an immediate infection and erosion after AUS implantation, and this patient ultimately had an AUS explantation. One NB patient had hydronephrosis and high pressure vesicoureteral reflux. We performed augmentation cystoplasty and concomitant ureteral reimplantation before AUS implantation. The patient had UUT function preserved with appropriate manipulation of the device (Fig. 1). AUS implantation is usually coupled with specific complications in NB patients, leading to higher re-operative rate than non-neurogenic patients. We recommend performing a staged procedure, confirming stable UUT function and no urethral stricture recurrence at least 6-month before AUS implantation, especially in complex reconstruction cases.

It has been reported that 30%–40% of patients who undergo prostatectomies complain of persistent PPI [11]. Approximately 2%–5% of patients with PPI exhibit persistent incontinence for at least 1 year post-operatively, despite conservative therapy attempts [12]. The incidence of PPI cases remains high despite advances in surgical technologies and techniques [13]. The minor percentage of PPI cases in our report may reflect the relatively small number of RP performed in China compared to the United States, and a difference of referral pattern to our center. Based on our experience, some patients with PPI may undergo improvement of continence status to an acceptable extent over time; other patients prefer seeking treatment if the status worsens. A recent study concluded that preserving membranous urethral length, depth of the urethrovesical junction, and nerve were related to the recovery time and level of urinary continence after RP [14]. Petroski. et al. [15] reported that UI can improve for up to 24 months after RP and early radiotherapy (RT) may interfere with or prolong return to continence. UI was more common in the early RT group and UI rates gradually improved over 3 years post-RT. In the same study, only 12 patients (26%) had an AUS placed. The process of PPI improvement should be considered when making a decision in terms of AUS placement for such patients.

The relatively higher rates of complications in the initial few patients (up to patient 7) may be a reflection of the learning curve needed to perfect the surgical techniques [16]. Previous urethral damage (failed surgical procedures and urethral atrophy) can potentially result in technical difficulties and/or reduce the efficacy of AUS surgery [17]. Most patients presenting with an AUS infection will have underlying cuff erosion [18]. In the present study, infection currently developed in approximately 12.5% of our patients. Cuff erosion occurred early post-operatively (patient 4) due to infection and later after convalescence (patients 5, 6, and 7) due to urethral damage secondary to cuff pressure and improper catheterization. Patient 5 had a revision, but ultimately experienced explantation due to infection. Patient 7 had recurrent SUI related to erosion and infection 36 months after the initial implantation. The previous cuff was removed with the remainder of the device sealed in vivo and transcorporal implantation was performed 6 months later. A recent study showed urethral repair at the time of explantation for cuff erosion appears to prevent stricture development, thus facilitating successful replacement [19]. We prefer a staged procedure allowing a period for healing after explantation, since infection and erosion often coexist. An aggressive repair may worsen urethra condition.

The transcorporal approach has been described as salvage surgical technique in patients with a damaged or frail urethra [17]. Noticeably, the transcorporal-implanted patient experienced descending efficacy due to the refractory UI. An imaging study showed urethral atrophy at the 6-year visit. Urethral atrophy is a common cause of recurrence UI during follow-up with a functioning AUS [13]. Urethral atrophy may result secondary to chronic compression of the urethra and urethral tissue hypoxia.

In our series, two patients (7 and 16) had previous male sling surgery, but with unsatisfactory outcomes; they finally received AUS implantation. Although there is insufficient long-term efficacy data on the male sling, most patients with moderate incontinence would choose a male sling and cite the primary reason being a motivation to avoid a mechanical device [20]. The sling may be preferable as an initial procedure because an AUS can be attempted after sling failure [21]. Generally, it is accepted that a convenient male sling could be an option for mild-to-moderate SUI, while AUS remains the gold standard treatment for severe SUI cases.

A potential weakness of this study was the relatively small sample, leading to a lack of power to detect subtle associations. The relatively higher rates of complications and differences in the frequency of causes may be attributable to the variety of etiologies for UI, complex conditions and combination surgeries. It is noteworthy that long-term follow-up and UUT monitoring is essential in special populations.

## Conclusion

The surgical management for complex UTR in UI cases including both neurogenic and non-neurogenic etiologies can be difficult. The present study indicated that AUS is a key procedure of UTR. The modalities of management must tailor to the unique needs of each individual. Appropriate patient counseling and adherence to surgical principles are vital for the success of surgery.

## Abbreviations

AUS: Artificial urinary sphincter
DSD: Detrusor sphincter dyssynergia
ISD: Intrinsic sphincter deficiency
MRU: Magnetic resonance urography
NB: Neurogenic bladder
PFUI: Pelvic fracture-associated urethral injuries
PPI: Post-prostatectomy incontinence
QoL: Quality of life
RI: Recurrent incontinence
RP: Post-radical prostatectomy
RT: Radio therapy
SUI: Stress urinary incontinence
UI: Urinary incontinence
UTR: Urinary tract reconstruction
UUT: Upper urinary tract
VAS: Visual analogue scale
